# Higher fatty liver index is associated with increased risk of new onset heart failure in healthy adults: a nationwide population-based study in Korea

**DOI:** 10.1186/s12872-020-01444-x

**Published:** 2020-04-28

**Authors:** Jae-Hyung Roh, Jae-Hyeong Park, Hanbyul Lee, Yong-Hoon Yoon, Minsu Kim, Yong-Giun Kim, Gyung-Min Park, Jae-Hwan Lee, In-Whan Seong

**Affiliations:** 1 Department of Cardiology in Internal Medicine, School of Medicine, Chungnam National University, Chungnam National University Hospital, 282 Munhwa-ro, Jung-gu, Daejeon, 35015 Korea; 2 Department of Statistics, Kyungpook National University, Daegu, Korea; 3 Department of Cardiology, Ulsan University Hospital, University of Ulsan College of Medicine, Ulsan, Korea

**Keywords:** Fatty liver index, Non-alcoholic fatty liver disease, Heart failure, Healthy people programs

## Abstract

**Background:**

Heart failure (HF) is relatively common cardiovascular disease with high mortality and morbidity. Although it is associated with many cardiovascular risk factors, the association between nonalcoholic fatty liver disease (NAFLD), the most common chronic liver disease, and HF has not been evaluated in a large-scale cohort study. Thus, we evaluated the ability of the fatty liver Index (FLI), a surrogate marker of NAFLD, to predict the development of HF in healthy individuals.

**Methods:**

We analyzed the association between the FLI and new-onset HF with multivariate Cox proportional-hazards models in 308,578 healthy persons without comorbidities who underwent the National Health check-ups in the republic of Korea from 2009 to 2014.

**Results:**

A total of 2532 subjects (0.8%) were newly diagnosed with HF during the study period (a median of 5.4 years). We categorized our subjects into quartile groups according to FLI (Q1, 0–4.9; Q2, 5.0–12.5; Q3, 12.6–31.0; and Q4, > 31.0). The cumulative incidence of HF was significantly higher in the highest FLI group than in the lowest FLI group (Q1, 307 [0.4%] and Q4, 890 [1.2%]; *P* < 0.001). Adjusted hazard ratio (HRs) indicated that the highest FLI group was independently associated with an increased risk for HF (HR between Q4 and Q1, 2.709; 95% confidence interval = 2.380–3.085; P < 0.001). FLI was significantly associated with an increased risk of new-onset HF regardless of their baseline characteristics.

**Conclusions:**

Higher FLI was independently associated with increased risk of HF in a healthy Korean population.

## Background

Non-alcoholic fatty liver disease (NAFLD) is the most common form of chronic liver disease. Its estimated prevalence is about 15–30% in general population [1]. The prevalence of NAFLD has been increasing along with the increase of obesity, insulin resistance and non-insulin dependent diabetes mellitus, and metabolic syndrome [2]. NAFLD can be found in about 60–70% among obese or diabetic population [1]. There are many studies showing associations between NAFLD and established cardiovascular risk factors including abdominal obesity, dyslipidemia, insulin resistance and high blood pressure [3]. These cardiovascular risk factors are also components of metabolic syndrome. Thus, NAFLD is regarded as the hepatic manifestation of metabolic syndrome. Moreover, cardiovascular disease is the most common cause of death in patients with NAFLD, besides extrahepatic malignancies and liver-related complications [4, 5].

Heart failure (HF) is one of major cardiovascular diseases with higher morbidity, mortality, and healthcare costs around the world [6, 7]. The prevalence of HF is approximately 1–2% of the adult population in developed countries and its prevalence is rising among aged people (≥10% among people > 70 years of age) [6, 8]. HF and NAFLD often coexist because they share the same risk factors and a similar pathophysiological processes [9, 10]. HF may cause liver disease, and liver disease may leads to HF in the absence of other cardiovascular risk factors [10].

Although there are several studies with small sample size showing the co-existence of HF and NAFLD [11, 12], they showed the simple association in several cross-sectional studies. We analyzed a large cohort consisting of a healthy Korean adult population without known traditional cardiovascular risk factors and co-morbidities to identify the association of NAFLD and new onset HF.

## Methods

### Data sources

We analyzed National Health Insurance Service-National Sample Cohort 2.0 (NHIS-NSC 2.0) data set. The Korean government has an obligatory public health insurance program, National Insurance Health Service (NIHS), including more than 97% of Korean people are affiliated. Total population (*n* = 48,222,537) were classified into 2142 classes according to their age, sex, area, eligibility status, and income level. Then, Korean government randomly selected 2.1% of them from each stratum (*n* = 1,021,208) from 2006 and made NHIS-NSC 2.0. It also included retrospective and prospective follow-up data which was collected from 2002 to 2015 [13]. Because the NHIS covers about 97% of the total population of Korea, the NHIS-NSC 2.0 cohort is expected to represent the entire Korean population.

The cohort includes 4 datasets: the dataset of the sociodemographic information; the dataset of medical claims including information on the diagnosis based on the 10th revision of the International Classification of Disease (ICD-10) codes, admission, and treatment; the dataset of the National Health Screening of the cohort members; and the dataset of the medical institutions. The Korean government recommends the entire Korean adults to take the National Health check-up biennially including questionnaires on medical history and health-related behaviors including smoking status and alcohol consumption, chest X-ray, physical examinations and blood tests. About 72.1% of eligible population had National Health screening programs according to the 2013 NHIS statistics [13]. The cohort also includes mortality data from the death registration database of the Statistics Korea, a central government organization for statistics.

The NHIS-NSC 2.0 is open to any researchers if the NHIS review committee approves study protocol. This study was approved by the Institutional Review Board of the Chungnam National University Hospital, Daejeon, Korea (IRB No. 2019–10-053). Our IRB waived the requirement for informed consent.

### Study population

We included population over 20 years of age having National Health check-ups at least one time from 2009 to 2014. We regarded the data from the first check-up as the index data, and the year of the index check-up as the index year. We included all subjects with age ≥ 20 years old and excluded all subjects having pre-specified exclusion criteria. We excluded all patients previously diagnosed with HF. To assess the effect of FLI on the new-onset HF, other exclusion criteria included comorbid conditions that can affect the onset of HF including hypertension, diabetes, atrial fibrillation, cerebrovascular disease, ischemic heart disease, peripheral vascular disease, valvular heart disease, chronic kidney disease, and chronic pulmonary disease. We excluded subjects with prescribed medications including oral hypoglycemic, antihypertensive, or lipid-lowering agents within 1 year before the index check-up, subjects with increased blood pressure level above the criteria of hypertension, and elevated fasting blood glucose ≥126 mg/dL at the index check-up. We also excluded factors that can affect FLI including liver disease, and autoimmune disease before the index year. Finally, those with missing data in the index check-up were also excluded.

### Definition of HF

The primary outcome of this study was HF incidence according to FLI, and the incidence of HF was defined as the first occurrence during at least 2 different days of hospital visits, at HF admission, or death with a diagnosis of HF. We assessed each diagnosis based on the data from questionnaires, and the 1-year claim data before the index year. When we used the claims data, we defined each diagnosis as the first occurrence during at least two different days of hospital visits (outpatient) or on the first admission, as likely a diagnosis of HF. The presence of HF was defined as those with “HF” according to the ICD-10 disease code in the claim dataset. HF patients who were assigned the following ICD-10 disease codes were considered as: “hypertensive heart disease with HF” (I10.0), “hypertensive heart disease with hypertensive kidney disease with HF” (I13.0),“hypertensive heart disease with hypertensive kidney disease with HF and kidney failure” (I13.2), “ischemic cardiomyopathy” (I25.5), “dilated cardiomyopathy” (I42.0), “cardiomyopathy, unspecified” (I42.9), “cardiomyopathy in diseases classified elsewhere” (I43), “HF” (I50) including “congestive HF” (I50.0), “left ventricular failure” (I50.1), and “HF, unspecified” (I50.9).

### Definition/ascertainment of covariates

We calculated body mass index (BMI) with dividing weight (kg) by height (m)-squared. Population with a BMI of ≥25 kg/m^2^ was regarded as obesity according to the World Health Organization guideline for the Asian population [14]. Smoking status was classified into 3 categories: non-smoker, ex-smoker, and current smoker. Alcohol consumption was evaluated with using standardized self-reporting questionnaires. The questionnaires about alcohol consumption were composed of the questions asking the number of days a week alcohol is consumed, and the amount of alcohol consumed on each drinking day. The amount of alcohol consumption was calculated by multiplying them. The questionnaires about physical activity were composed of the questions asking the number of days a week 30 min of light exercise, 30 min of moderate exercise, and 20 min of vigorous exercise are performed, respectively. Light exercise was assumed to be 2 METs, moderate 3 METs, and vigorous 6 METs, which were multiplied by 20, 30, and 30 min and the respective number of days a week, and summed.

Considering the nation-wide scale of this study, laboratory test cannot be performed in a central facility. Instead, blood samples were analyzed in a number of different institutions which were qualified by an external quality assessment service, annually conducted by Korean Association of External Quality Assessment Service. Data were censored at the time of HF occurrence, disqualification of the NHIS (death or immigration), or the end of the study (December 31th, 2015).

### Calculation fatty liver index

We used a well validated, surrogate marker, FLI to identify patients with NAFLD [13]. FLI was calculated with 4 variables (triglycerides [TG], BMI, gamma-glutamyl transferase [GGT], and waist circumference [WC]) with following equation:

$$ \mathrm{FLI}=\left({\mathrm{e}}^{0.953\times \log }{{{{}_{\mathrm{e}}}^{\left(\mathrm{TG}\right)+0.139\times BMI+0.718\times \log}}_{\mathrm{e}}}^{\left(\mathrm{GGT}\right)+0.053\times \mathrm{WC}\hbox{-} 15.745}\right)/\left(1+{\mathrm{e}}^{0.953\times \log }{{{{}_{\mathrm{e}}}^{\left(\mathrm{TG}\right)+0.139\times \mathrm{BMI}+0.718\times \log}}_{\mathrm{e}}}^{\left(\mathrm{GGT}\right)+0.053\times \mathrm{WC}\hbox{-} 15.745}\right)\times 100 $$

The original study showed that the FLI more than 60 as the cutoff for the diagnosis of fatty liver with positive likelihood ratio of 4.3 in general population [15]. Although the FLI is simple to calculate, and easy to screen fatty liver disease, there has been insufficient evidence regarding the diagnosis of fatty liver disease with FLI in Asians because of lower BMI and WC than other ethnic population [16]. Thus, we categorized our study group into quartiles according to their FLI and used quartile group in the statistical analysis.

### Statistical analysis

We used continuous variables as mean ± standard deviation and categorical parameters as number with percentage. We performed all statistical analysis with using R software version 3.3.3 (R Foundation for Statistical Computing, Vienna, Austria; www.r-project.org). Chi-square test and one-way analysis of variance test were used to evaluate statistical differences among the FLI quartiles. The calculation of cumulative event rates according to the FLI quartiles was done with Kaplan-Meier method and compared with a log-rank test. Adjusted hazard ratios (HR) and 95% confidence interval (CI) for HF incidence were estimated with Cox proportional hazard regression analysis. In the multivariate analysis, we adjusted age and sex in the model 1, and clinical characteristics associated with new onset HF of borderline statistical significance (*P* < 0.100) along with age and sex in the model 2. We excluded several confounding factors such as hypertension and diabetes because they have significant associations with NAFLD. The inclusion of NAFLD along with these risk factors in the multivariate model might have introduced multicollinearity into the model. We checked the multicollinearity issue by checking the variance inflation factor (VIF) in all models (Supplementary Table 1). Because the VIF levels in the models were less than 10, there was no multicollinearity issue in the models. *P* values of < 0.05 were considered statistically significant.

## Results

### Baseline characteristics of the participants

**Fig. 1:**
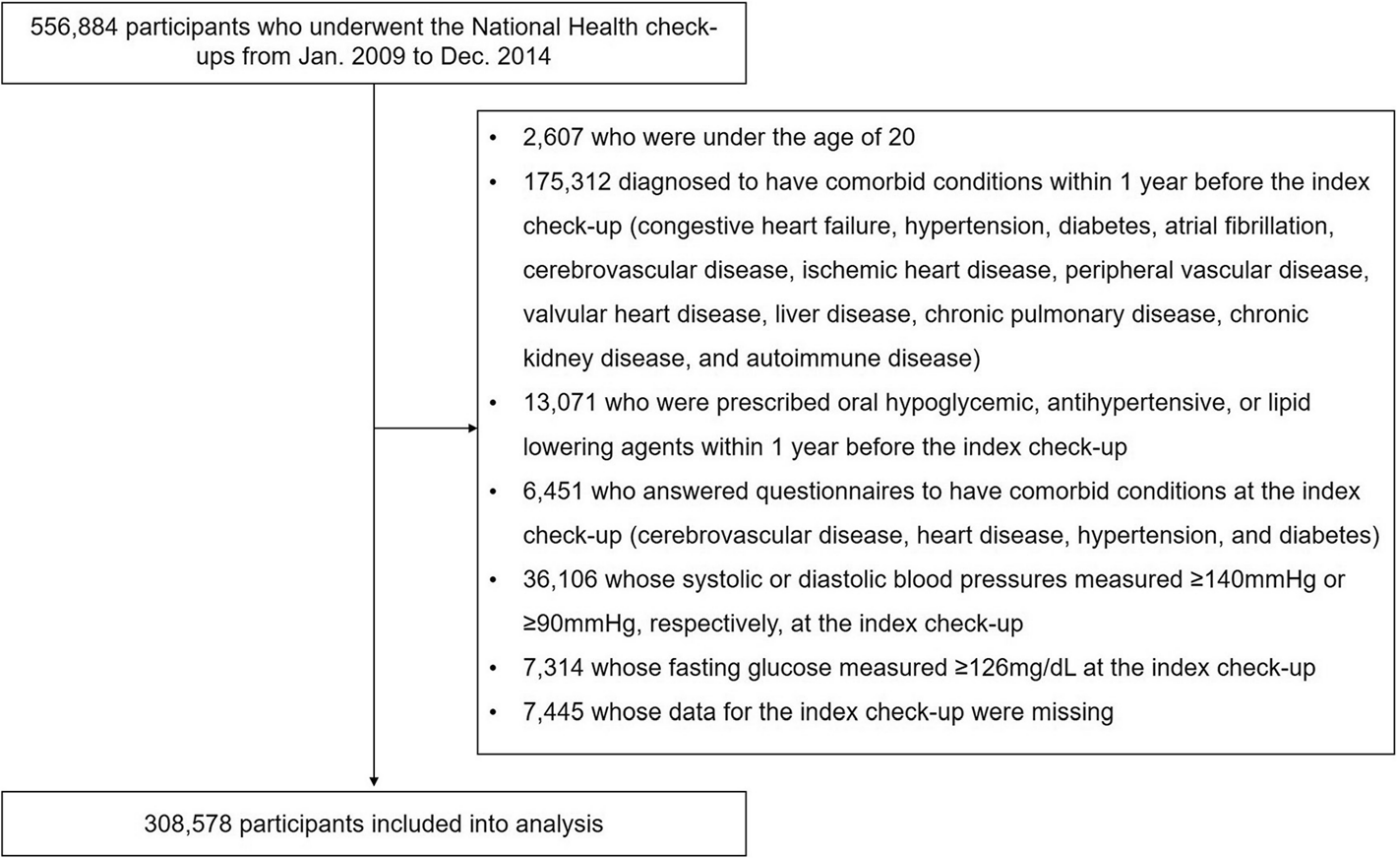
Overview of the study population

We analyzed 308,578 subjects after the exclusion of 248,306 subjects who having the pre-specified exclusion criteria. The number of the subjects matching each exclusion criteria was presented in the Fig. 1. We divided our study population into 4 groups according to their FLI quartile values; first quartile (Q1), 0–4.9; second quartile (Q2), 5.0–12.5; third quartile (Q3), 12.6–31.0; and fourth quartile (Q4), > 31.0. The comparison of baseline clinical characteristics and laboratory findings according to the FLI quartiles was summarized in the Table 1. The subjects with higher FLI had a trend to higher age and higher incidence of male. BMI, waist circumference, blood pressures, the amount of alcohol consumption and the proportion of current smoker tended to increase from Q1 to Q4. Fasting glucose, total cholesterol, triglyceride and LDL cholesterol levels were increasing along with the increase of FLI quartiles.

**Table 1:** Baseline characteristics and laboratory findings

	FLI quartile				*P*-value	P for trend
	Q1	Q2	Q3	Q4		
**Baseline characteristics**						
Age, years	36.7 ± 11.4	41.9 ± 12.4	44.0 ± 12.4	43.1 ± 11.3	< 0.001	< 0.001
Male sex (%)	12,102 (15.7)	30,737 (39.8)	46,692 (60.5)	62,507 (81.0)	< 0.001	< 0.001
Height, cm	161.4 ± 7.2	163.4 ± 8.9	165.6 ± 9.3	168.8 ± 8.4	< 0.001	< 0.001
Weight, Kg	52.2 ± 6.1	59.1 ± 7.3	65.4 ± 8.3	75.0 ± 10.4	< 0.001	< 0.001
BMI, Kg/m^2^	20.0 ± 1.7	22.1 ± 1.8	23.8 ± 2.1	26.3 ± 2.8	< 0.001	< 0.001
Waist circumference, cm	67.9 ± 4.9	74.9 ± 4.8	80.5 ± 5.1	87.6 ± 6.5	< 0.001	< 0.001
WHR, %	0.3 ± 0.0	0.4 ± 0.0	0.4 ± 0.0	0.4 ± 0.1	< 0.001	< 0.001
SBP, mmHg	111.0 ± 11.1	115.4 ± 11.1	118.6 ± 10.6	121.8 ± 10.0	< 0.001	< 0.001
DBP, mmHg	69.4 ± 8.0	72.0 ± 7.9	74.0 ± 7.6	76.3 ± 7.1	< 0.001	< 0.001
Smoking					< 0.001	< 0.001
Non-smoker	65,184 (84.5)	53,779 (69.7)	42,729 (55.4)	28,355 (36.8)		
Ex-smoker	3609 (4.7)	6720 (8.7)	10,711 (13.9)	14,058 (18.2)		
Current-smoker	8350 (10.8)	16,638 (21.6)	23,713 (30.7)	34,732 (45.0)		
Alcohol consumption						
Amount, g/week	31.6 ± 77.4	49.5 ± 102.2	73.9 ± 129.6	122.5 ± 174.0	< 0.001	< 0.001
Activity, MET-min/week	346.7 ± 350.8	379.7 ± 382.7	388.8 ± 388.1	374.6 ± 373.4	< 0.001	< 0.001
**Laboratory findings**						
AST, IU/L	19.8 ± 8.0	21.7 ± 12.4	23.7 ± 16.7	29.4 ± 33.0	< 0.001	< 0.001
ALT, IU/L	14.5 ± 8.9	17.9 ± 12.2	22.9 ± 22.2	36.3 ± 50.5	< 0.001	< 0.001
GGT, IU/L	14.2 ± 5.6	19.1 ± 9.9	28.1 ± 20.3	60.3 ± 62.4	< 0.001	< 0.001
Hemoglobin, g/dL	13.0 ± 1.4	13.6 ± 1.6	14.2 ± 1.6	14.9 ± 1.4	< 0.001	< 0.001
Fasting glucose, mg/dL	87.9 ± 9.5	90.3 ± 10.2	92.2 ± 10.7	94.8 ± 11.4	< 0.001	< 0.001
Total cholesterol, mg/dL	176.9 ± 33.6	187.1 ± 35.9	196.2 ± 39.3	207.0 ± 40.7	< 0.001	< 0.001
Triglyceride, mg/dL	62.6 ± 22.5	88.0 ± 34.0	119.9 ± 53.6	197.6 ± 132.6	< 0.001	< 0.001
HDL cholesterol, mg/dL	64.1 ± 24.8	59.3 ± 19.9	55.5 ± 25.8	51.7 ± 32.0	< 0.001	< 0.001
LDL cholesterol, mg/dL	109.4 ± 257.2	113.6 ± 143.5	121.2 ± 134.2	122.6 ± 129.0	< 0.001	< 0.001
Creatinine, mg/dL	0.9 ± 1.0	1.0 ± 1.1	1.0 ± 1.1	1.1 ± 1.2	< 0.001	< 0.001
GFR, mL/min/1.73m^2^	90.2 ± 27.9	90.3 ± 28.0	92.6 ± 29.4	99.7 ± 30.2	< 0.001	< 0.001

*ALT* alanine aminotransferase, *AST* aspartate aminotransferase, *BMI* body mass index, *DBP* diastolic blood pressure, *GFR* glomerular filtration rate, *GGT* gamma-glutamyl transferase, *HDL* high-density lipoprotein, *LDL* low-density lipoprotein, *SBP* systolic blood pressure, *WHR* waist-hip ratio

### Association between FLI and the incidence of HF

**Fig. 2:**
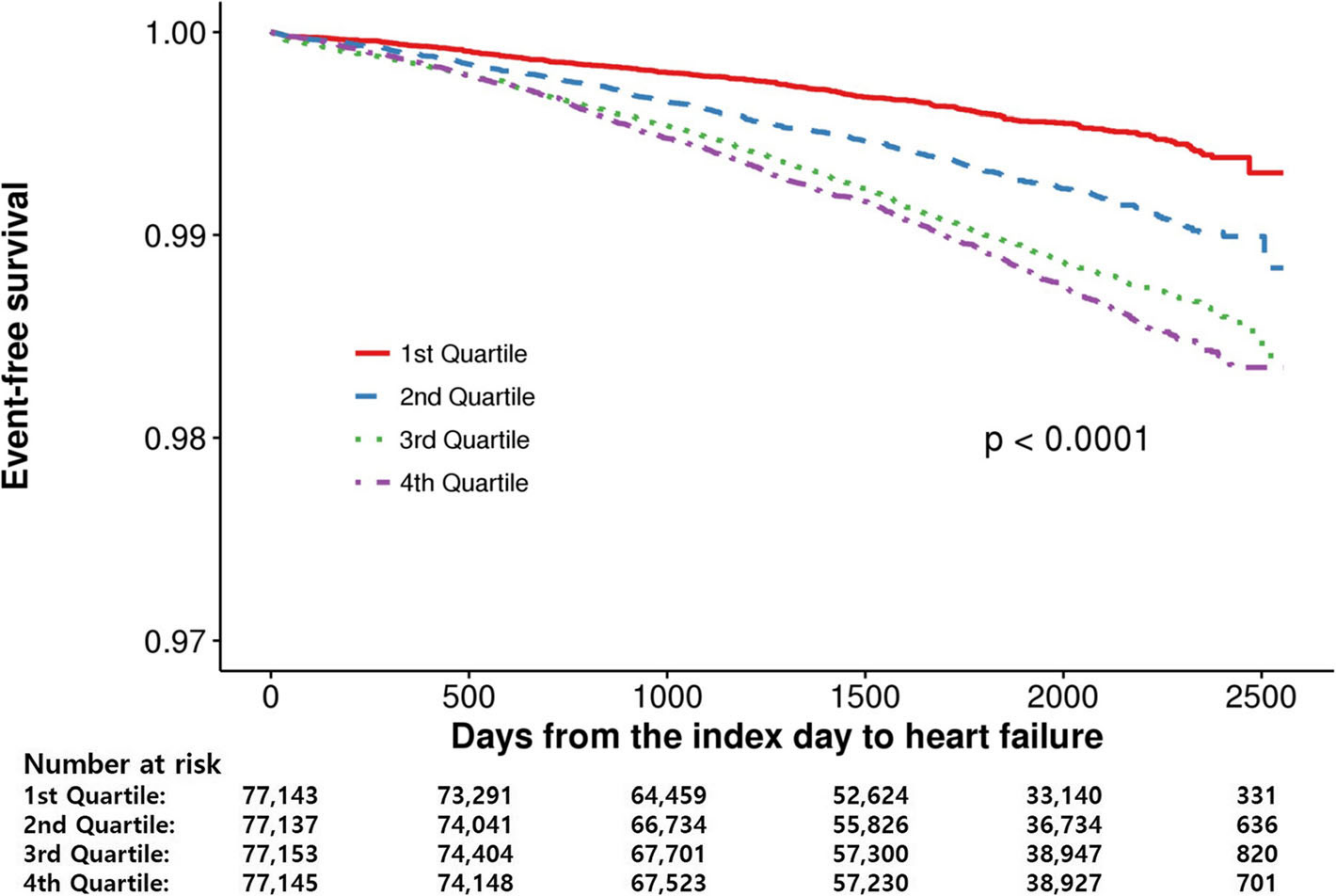
Cumulative incidence of new onset heart failure according to their quartiles. Statistical significance was determined using the log-rank test

The total follow-up duration of this study cohort was median 5.4 years (interquartile range, 4.1–6.3), 2532 subjects (0.8%) had new onset HF. Table 2 shows the result of univariate analysis of new onset HF. Male gender, older age, higher BMI, higher total cholesterol, TG, and lower HDL cholesterol were associate significantly with new onset HF. Cumulative incidences of new onset HF according to the FLI quartiles were presented in the Fig. 2. The incidence of HF was significantly higher in the subjects with higher FLIs, compared to those with lower FLIs (Q1, 307 [0.4%]; Q2, 543 [0.7%]; Q3, 792 [1.0%]; and Q4, 890 [1.2%], *P* < 0.001 by the Log-rank test). In multivariate models which were adjusted for age and sex in the model 1 and for clinical characteristics with borderline statistical significance along with age and sex in the model 2, the association between FLI quartile and HF incidence remained statistically significant in each model in the time-dependent Cox proportional hazard analsysis (Table 3). When we used various cutoff-points were tried to categorize the study population, which were suggested by the previous studies (0 ≤ FLI < 30, 30 ≤ FLI < 60, and FLI ≥ 60, by Bedogni et al. [15]; 0 ≤ FLI < 25, 25 ≤ FLI < 35, and FLI ≥ 35 for male, 0 ≤ FLI < 10, 10 ≤ FLI < 20, and FLI ≥ 20 for female, by Yang et al. [17]). Regardless of the cutoff-points used, the highest FLI group had the highest risk for new onset HF (Supplementary Table 2).

**Table 2:** Univariable analysis of new-onset heart failure

Characteristics	Value	N	HF (%)	HR	95% CI		P-value
					Lower	Upper	
Total	Total	308,578	2532 (0.8)				
Sex	Female	156,540	1263 (0.8)	Reference			
	Male	152,038	1269 (0.8)	1.113	1.030	1.204	0.007
Age, year	20–34	99,774	119 (0.1)	Reference			
	35–49	130,099	637 (0.5)	3.908	3.213	4.753	< 0.001
	50–64	65,907	1067 (1.6)	12.536	10.371	15.153	< 0.001
	65–74	10,374	497 (4.8)	35.791	29.298	43.722	< 0.001
	≥75	2424	212 (8.7)	75.652	60.429	94.711	< 0.001
	Continuous			1.087	1.084	1.090	< 0.001
BMI, Kg/m^2^	< 18.5	16,662	101 (0.6)	1.075	0.870	1.328	0.505
	18.5–19.9	33,181	177 (0.5)	0.929	0.786	1.098	0.390
	20–22.4	89,515	625 (0.7)	Reference			
	22.5–24.9	90,961	814 (0.9)	1.115	1.005	1.238	0.041
	≥25	78,259	815 (1.0)	1.417	1.276	1.573	< 0.001
	Continuous			1.058	1.045	1.072	< 0.001
Smoking	Non-smoker	190,047	1599 (0.8)	Reference			
	Ex-smoker	35,098	320 (0.9)	0.934	0.812	1.074	0.340
	Current-smoker	83,433	613 (0.7)	1.120	0.997	1.259	0.057
Alcohol consumption, g/week	0	149,779	1557 (1.0)	Reference			
	<=140	110,537	626 (0.6)	0.882	0.798	0.975	0.014
	> 140	48,262	349 (0.7)	0.991	0.871	1.126	0.890
	Continuous			1.000	1.000	1.000	0.960
SBP, mmHg	< 120	165,193	1019 (0.6)	Reference			
	120–139	143,385	1513 (1.1)	1.295	1.195	1.404	< 0.001
	Continuous			1.013	1.010	1.017	< 0.001
DBP, mmHg	< 80	210,638	1513 (0.7)	Reference			
	80–89	97,940	1019 (1.0)	1.220	1.126	1.322	< 0.001
	Continuous			1.016	1.011	1.022	< 0.001
FBS, mg/dL	< 100	246,415	1876 (0.8)	Reference			
	100–125	62,163	656 (1.1)	1.042	0.953	1.140	0.370
	Continuous			1.003	1.000	1.007	0.058
Activity, MET-min/week	< 500	221,107	1871 (0.8)				
	500–999	65,361	453 (0.7)	0.877	0.791	0.972	0.013
	≥1000	22,110	208 (0.9)	0.890	0.770	1.027	0.110
	Continuous			1.000	0.999	1.000	0.210
Waist circumference, cm	Continuous			1.027	1.022	1.032	< 0.001
Total cholesterol, mg/dL	Continuous			1.001	1.000	1.002	< 0.001
Triglyceride, mg/dL	Continuous			1.001	1.000	1.001	< 0.001
HDL-cholesterol, mg/dL	Continuous			0.997	0.995	0.999	0.014
LDL-cholesterol, mg/dL	Continuous			1.000	1.000	1.000	0.290

*BMI* body mass index, *CI* confidential interval, *DBP* diastolic blood pressure, *FBS* fasting blood glucose, *HDL* high-density lipoprotein, *HF* heart failure, *HR* hazard ration, *LDL* low-density lipoprotein, *MET* metabolic equivalent of task, *SBP* systolic blood pressure

**Table 3:** Association between fatty liver index and new-onset heart failure

FLI	N.	New-onset HF	Univariate			Model 1^a^			Model 2^b^		
			HR	95% CI	*P*-value	HR	95% CI	*P*-value	HR	95% CI	*P*-value
FLI quartile											
Q1 (0–4.9)	77,143	307 (0.4)	Reference			Reference			Reference		
Q2 (5.0–12.5)	77,137	543 (0.7)	1.691	1.470–1.945	< 0.001	1.104	0.958–1.271	0.171	1.071	0.930–1.234	0.339
Q3 (12.6–31.0)	77,153	792 (1.0)	2.409	2.112–2.748	< 0.001	1.386	1.212–1.586	< 0.001	1.314	1.147–1.504	< 0.001
Q4 (> 31.0)	77,145	890 (1.2)	2.709	2.380–3.085	< 0.001	1.857	1.620–2.127	< 0.001	1.710	1.489–1.964	< 0.001

*CI* confidential interval, *FLI* fatty liver index, *HR* hazard ratio

^a^Cox proportional hazard models including age, and sex as covariates

^b^Cox proportional hazard models including age, sex, smoking, amount of alcohol drinking, activity, systolic blood pressure, diastolic blood pressure, fasting blood glucose, cholesterol and fatty liver index as covariates

### Subgroup analysis

**Fig. 3:**
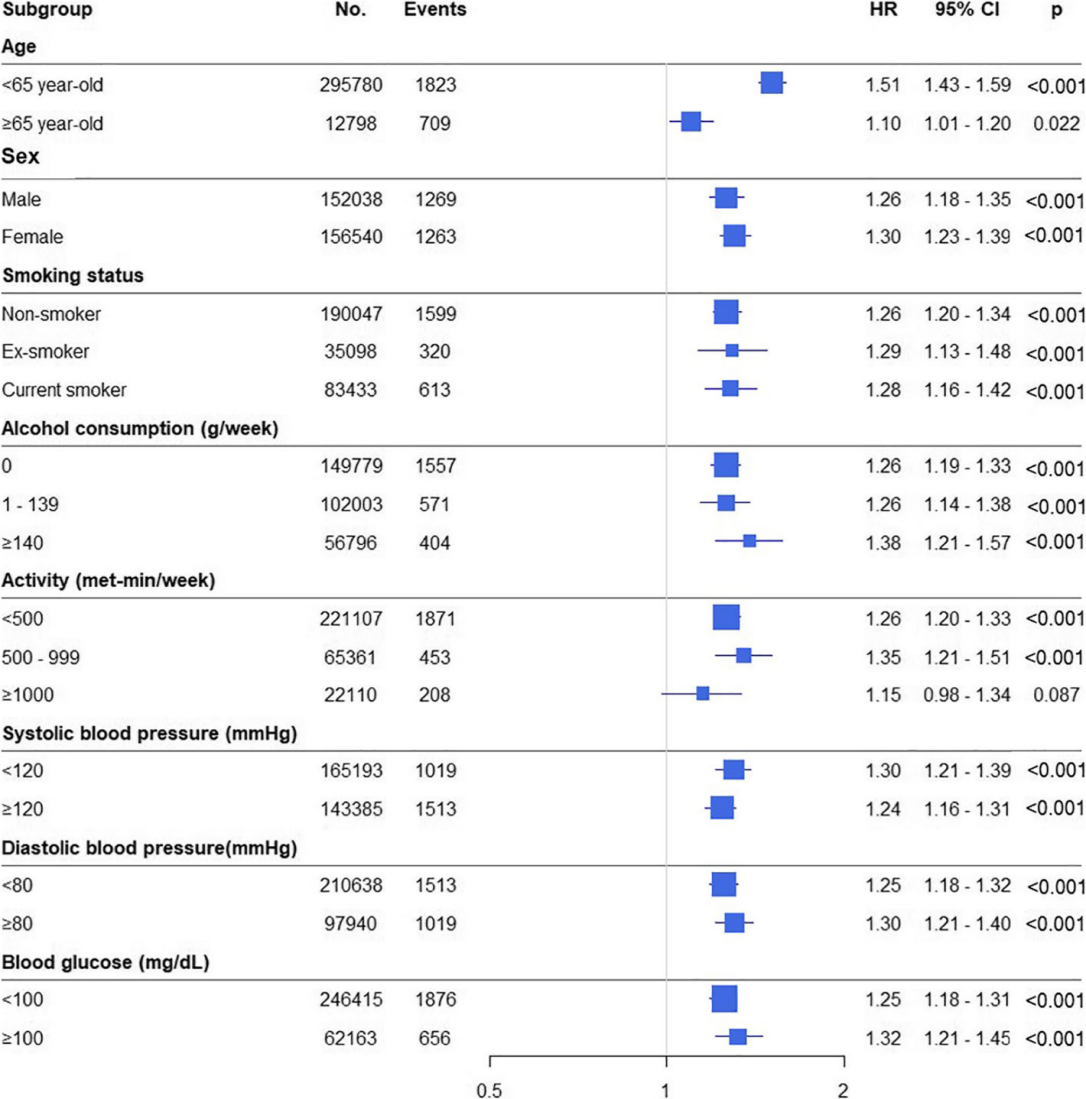
Forest plots of hazard ratios for new onset heart failure stratified by various clinical characteristics. CI = confidence interval; HR = hazard ratio

Adjusted HRs according to subgroups summarized in the Fig. 3. FLI was a significant determinant of new onset HF in all subgroups evaluated (Fig. 3).

## Discussion

In this study, we showed higher FLI, a well validated index of NAFLD, was associated with increased risk of new onset HF in healthy Korean adult population. In this study, we adopted multivariate models to control several confounding factors such as hypertension and diabetes, which are well-known risk factors for HF. We evaluated more direct associations between NAFLD and HF after exclusion of hypertension and diabetes form the multivariate models. This association was found in most of the subgroups.

The association between fatty liver and HF has been observed in several studies [11, 12], and mildly elevated serum GGT levels in the absence of excessive alcohol consumption were long-term, independent predictors of incident HF in some large population-based studies [18–20]. Zhang et al. reported 37 had NAFLD defined by ultrasonography in 102 patients with HF with reduced ejection fraction (HFrEF) (36.3%) [12]. In the Framingham study, higher GGT level, even within the normal range, had a 1.71-fold risk of HF (95% CI = 1.21–2.41) compared with participants with GGT concentrations less than the median [18]. Also, GGT improved the risk reclassification modestly (net reclassification index, 5.7%; *P* = 0.01) in the HF prediction [18]. In a prospective cohort study performed in Finland including 18,353 men and 19,726 women who were 25–74 years of age, moderate to high concentrations of serum GGT (from the 50th to the 90th percentiles) were significantly associated with incident HF, and the predictive power was stronger in participants aged < 60 years [20].

The presence of NAFLD was associated with higher BMI and left ventricular (LV) mass index, and had more severe LV fibrosis [12]. Other studies showed that patients with NAFLD had increased LV wall thickness and varying degrees of subclinical LV systolic or diastolic dysfunction [21, 22]. Increased LV wall thickness and fibrosis is a marker of LV diastolic dysfunction regardless of their symptoms. Jung et al. showed mild and moderate to severe NAFLD group had the higher risk of having abnormal LV relaxation compared with normal controls [21]. They showed increased odds ratios for abnormal LV relaxation [mild group: 1.29 (95% confidence interval: 1.15–1.46), moderate to severe group: 1.95 (95% confidence interval: 1.61–2.35)] and increased relative wall thickness (> 0.42) [mild group: 1.26 (95% confidence interval: 1.05–1.52), moderate to severe group: 1.46 (95% confidence interval: 1.08–1.95)] in their cohort having 20,821 Korean adults received health check-ups. The proposed mechanism of increased LV wall thickness include insulin resistance [23], increased renin-angiotensin-aldosterone system (RAAS) [24], and endothelial dysfunction and inflammation [11]. Insulin resistance may contribute to the development of LV hypertrophy and HF through increased renal sodium retention and activation of the sympathetic nervous system [25, 26]. Increased sympathetic nervous system can also increase hepatic fibrosis [27]. Mediators of RAAS, especially angiotensin II and aldosterone, play important roles in the development of hypertension and HF. Also, angiotensin II can be expressed by activated human hepatic stellate cells [24], and activated local RAAS increase hepatic injury and induce fibrosis through angiotensin II-mediated stimulation of fibroblast proliferation and increased release of inflammatory cytokines [28]. Endothelial dysfunction, as a component of inflammation, can increase arterial stiffness and LV afterload through increased vascular tone, sympathetic overactivity, and sodium retention [29]. Inflammation can promote coronary atherosclerosis and increase risk for cardiomyopathy and conduction abnormalities [11]. Inflammatory mediators also affect the progression of fatty liver disease via impaired fatty acid oxidation, increased oxidative stress, and local inflammation [30].

The presence of NAFLD was associated with poor prognosis of chronic HF [31] and acute HF [32]. Although mechanisms of the association between NAFLD and poor clinical outcomes of HF remains unclear, there are several proposed explanations including increased coronary atherosclerosis, enhancing LV hypertrophy and dysfunction, and increased aortic valve calcification [33–35]. Because liver plays an important role in the regulation of antioxidant and anti-inflammation systems [36], hepatic dysfunction can increase chronic inflammation and oxidative tissue injury in patient with HF [37] and the impaired antioxidant and antiinflammation roles of liver may further deteriorate the prognosis of HF patients [38].

### Limitations

Our study has several limitations. First, we analyzed only claims data and National Health Screening data without echocardiographic or chemical data. Moreover, we estimated new onset HF by using the NHIS-NSC 2.0 database, which is based on ICD-10 disease code, and the accuracy of HF diagnosis was not validated in this study. If we use echocardiographic data and serum biomarker including B-type natriuretic peptide or N terminal pro B-type natriuretic peptide, the diagnosis of HF would be accurate. Further studies should be needed to validate HF patients with health insurance data using either hospital-based medical records. Second, we demonstrated the simple relationship between increased FLI and increased new-onset HF and cannot show other contributable factors in the development of HF because this study is an observational study. Third, we excluded about 45% of the participants in the cohort. This could affect the representativeness and generalizability of our findings. Finally, this study population included only Koreans. Thus, caution should be needed in the generalization of this data to other ethnic populations.

## Conclusions

Our findings demonstrated that higher FLI was independently associated with increased risk for HF in healthy Korean population.

### Clinical perspectives

Because control of metabolic syndrome and prevention of HF are important in the reduction of cardiovascular morbidity and mortality, the identification of increased HF risk may have important public health implications. Further studies are needed to document the effect of management of NAFLD not only on the risk of new onset HF, but also on the reduction of cardiovascular morbidity and mortality.

## Supplementary information

**Additional file 1 **Supplementary **Table 1**. Variance inflation factor (VIF) in all models.

**Additional file 2 **Supplementary **Table 2** Association between fatty liver index and new-onset heart failure.

## Data Availability

The data that support the findings of this study are available from the National Health Insurance Service of Korea but restrictions apply to the availability of these data, which were used under license for the current study, and so are not publicly available. Data are however available from the authors upon reasonable request and with permission of the National Health Insurance Service of Korea.

## Abbreviations

BMI: Body mass index
FLI: Fatty liver index
CI: Confidence interval
GGT: Gamma-glutamyl transferase
HF: Heart failure
HR: Hazard ratios
ICD: International classification of disease
LV: Left ventricle
METs: Metabolic equivalent of task
NAFLD: Non-alcoholic fatty liver disease
NHIS: National Health Insurance Service
NHIS-NSC: National Health Insurance Service-National Sample Cohort
RAAS: Renin-angiotensin-aldosterone system
TG: Triglycerides
VIF: Variance inflation factor
WC: Waist circumference
