# Supplementary material for: Higher fatty liver index is associated with increased risk of new onset heart failure in healthy adults: a nationwide population-based study in Korea
Source: BMC Cardiovasc Disord. 2020 Apr 28;20:204. doi: 10.1186/s12872-020-01444-x (PMC7189566; doi:10.1186/s12872-020-01444-x)
Supplement: Supplementary file 1 — Additional file 1 Supplementary Table 1. Variance inflation factor (VIF) in all models. [file 12872_2020_1444_MOESM1_ESM.docx]

Supplementary table 1. Variance inflation factor (VIF) in all models.

| Model 1* | | Model 2† | |
| --- | --- | --- | --- |
| Variable | VIF | Variable | VIF |
| Age | 1.044 | Age | 1.064 |
| Sex | 1.161 | Sex | 1.162 |
| FLI | 1.060 | Smoking | 1.072 |
|  |  | Drinking | 1.102 |
|  |  | Activity | 1.004 |
|  |  | Systolic BP | 1.460 |
|  |  | Diastolic BP | 1.437 |
|  |  | FBS | 1.046 |
|  |  | Total cholesterol | 1.001 |
|  |  | FLI | 1.060 |

BP: blood pressure; FBS: fasting blood sugar; FLI: fatty liver index

^*^Cox proportional hazard models including age, and sex as covariates

^†^Cox proportional hazard models including age, sex, smoking, amount of alcohol drinking, activity, systolic blood pressure, diastolic blood pressure, fasting blood glucose, cholesterol and fatty liver index as covariates.
