# Supplementary material for: Higher fatty liver index is associated with increased risk of new onset heart failure in healthy adults: a nationwide population-based study in Korea
Source: BMC Cardiovasc Disord. 2020 Apr 28;20:204. doi: 10.1186/s12872-020-01444-x (PMC7189566; doi:10.1186/s12872-020-01444-x)
Supplement: Supplementary file 2 — Additional file 2 Supplementary Table 2 Association between fatty liver index and new-onset heart failure. [file 12872_2020_1444_MOESM2_ESM.docx]

Supplementary table 2. Association between fatty liver index and new-onset heart failure

| FLI | N. | New-onset HF | Univariate | | | Model 1* | | | Model 2^†^ | | |
| --- | --- | --- | --- | --- | --- | --- | --- | --- | --- | --- | --- |
|  |  |  | HR | 95% CI | P-value | HR | 95% CI | P-value | HR | 95% CI | P-value |
| FLI criteria 1 |  |  |  |  |  |  |  |  |  |  |  |
| 0 ≤ FLI < 30 | 228,136 | 1,608(0.7) | Reference |  |  | Reference |  |  | Reference |  |  |
| 30 ≤ FLI < 60 | 54,563 | 614(1.1) | 1.538 | 1.402-1.688 | <0.001 | 1.376 | 1.250-1.514 | <0.001 | 1.320 | 1.198-1.453 | <0.001 |
| FLI ≥ 60 | 25,879 | 310(1.2) | 1.669 | 1.478-1.885 | <0.001 | 1.961 | 1.727-2.227 | <0.001 | 1.849 | 1.622-2.106 | <0.001 |
| FLI criteria 2‡ |  |  |  |  |  |  |  |  |  |  |  |
| Low probability | 178,464 | 10,20(0.6) | Reference |  |  | Reference |  |  | Reference |  |  |
| Intermediate probability | 46,892 | 480(1.0) | 1.735 | 1.556-1.933 | <0.001 | 1.233 | 1.105-1.376 | <0.001 | 1.196 | 1.072-1.334 | 0.001 |
| High probability | 83,222 | 1,032(1.2) | 2.104 | 1.930-2.294 | <0.001 | 1.631 | 1.496-1.779 | <0.001 | 1.557 | 1.423-1.704 | <0.001 |

^*^Cox proportional hazard models including age, and sex as covariates

^†^Cox proportional hazard models including age, sex, smoking, amount of alcohol drinking, activity, systolic blood pressure, diastolic blood pressure, fasting blood glucose, cholesterol and fatty liver index as covariates.

^‡^Low probability: 0 ≤ FLI < 25 for male, 0 ≤ FLI < 10 for female; intermediate probability: 25 ≤ FLI < 35 for male, 10 ≤ FLI < 20 for female; high probability: FLI ≥ 35 for male, FLI ≥ 20 for female

CI: confidential interval, FLI: fatty liver index, HR: hazard ratio
